# GC-MS Studies on the Conversion and Derivatization of γ-Glutamyl Peptides to Pyroglutamate (5-Oxo-Proline) Methyl Ester Pentafluoropropione Amide Derivatives

**DOI:** 10.3390/molecules27186020

**Published:** 2022-09-15

**Authors:** Alexander Bollenbach, Dimitrios Tsikas

**Affiliations:** Core Unit Proteomics, Institute of Toxicology, Hannover Medical School, 30625 Hannover, Germany

**Keywords:** acylation, amidation, derivatization, esterification, γ-glutamyl peptides, glutathione, GC-MS, pentafluoropropionic anhydride, pyroglutamate

## Abstract

Glutathione (γ-L-glutamyl-L-cysteinyl-glycine, γ-Glu-Cys-Gly) is the most abundant intra-cellular dicarboxylic tripeptide with multiple physiological roles. In biological samples, glutathione exists in its reduced form GSH and in two stable oxidized forms, i.e., in its symmetric disulfide form GSSG and as *S*-glutathionyl residue in proteins. *S*-Glutathionylation is a post-translational modification, which is involved in several pathophysiological processes, including oxidative stress. The GSH-to-GSSG molar ratio is widely used as a measure of oxidative stress. γ-Glutamyl is the most characteristic structural moiety of GSH. We performed gas chromatography-mass spectrometry (GC-MS) studies for the development of a highly specific qualitative and quantitative method for γ-glutamyl peptides. We discovered intra-molecular conversion of GSH, GSSG, γ-Glu-Cys and of ophthalmic acid (OPH; γ-glutamyl-α-amino-*n*-butyryl-glycine) to pyroglutamate (pGlu; 5-oxo-proline, also known as pidolic acid) during their derivatization with 2 M HCl/CH_3_OH (60 min, 80 °C). For GC-MS analysis, the methyl esters (Me) were further derivatized with pentafluoropropionic (PFP) anhydride in ethyl acetate (1:4, *v*/*v*; 30 min, 65 °C) to their PFP derivatives. At longer reaction times, pGlu is hydrolyzed to Glu. Internal standards were prepared by derivatizing GSH, GSSG, γ-Glu-Cys and OPH in 2 M HCl/CD_3_OD. Quantification of the Me-PFP derivative of pGlu was performed in the electron-capture negative-ion chemical ionization (ECNICI) mode by selected-ion monitoring (SIM) of the mass-to-charge (*m*/*z*) ions 269 for unlabeled pGlu (d_0_Me-PFP-pGlu) and *m*/*z* 272 for the in situ prepared deuterium-labeled pGlu (d_3_Me-PFP-pGlu). Although not inherent to the analysis of small peptides, the present GC-MS method is useful to study several biochemical aspects of GSH. Using pentafluorobenzyl bromide (PFB-Br) as the derivatization reagent, we found that synthetic pGlu is converted in aqueous acetone (60 min, 50 °C) into its pentafluorobenzyl (PFB) ester (PFB-pGlu). This derivatization procedure is useful for the GC-MS analysis of free pGlu in the ECNICI mode. Quantitative analysis of PFB-pGlu by GC-MS requires the use of stable-isotope labeled analogs of pGlu as an internal standard.

## 1. Introduction

Glutathione (GSH, γ-L-glutamyl-L-cysteinyl-glycine; γ-Glu-Cys-Gly) is the most abundant endogenous low-molecular-mass thiol, virtually present in all types of animals, plants, fungi, and some bacteria and archaea [1,2,3]. GSH is a tripeptide with a γ-peptide linkage between the carboxyl group of the Glu side chain and the amine group of the Cys moiety. The carboxyl group of the Cys residue is attached by normal peptide linkage to Gly. In plants, fungi, nematodes and all groups of algae, including cyanobacteria, GSH occurs as oligomers in phytochelatins (PC), i.e., (γ-Glu-Cys)*_n_*-Gly (*n* = 2 to 11), which mainly act as chelators of heavy metal ions [4].

Glutamate cysteine ligase (GCL; EC 6.3.2.2), also known as γ-glutamyl-cysteine synthetase (GCS), is the first enzyme in the cellular biosynthetic pathway of GSH [1,2,3]. GCS catalyzes the chemical reaction of L-Glu with L-Cys, thereby releasing inorganic phosphate (P_i_) (R1). Glutathione synthase (GSS; EC 6.3.2.3) is the second enzyme involved in the biosynthesis of GSH. GSS catalyzes the chemical reaction of γ-L-Glu-L-Cys with Gly (R2) to produce γ-L-Glu-L-Cys-Gly (i.e., GSH). GCS and GSS use adenosine triphosphate (ATP) as a co-substrate. GSH plays multiple important physiological roles. For example, GSH is a cofactor and substrate for many enzymes, including GSH *S*-transferase and leukotriene C_4_ synthase. GSSG is a substrate for glutathione reductase (EC 1.8.1.7), which reduces GSSG to GSH [1,2,3].

L-Glu + L-Cys + ATP ⇌ γ-L-Glu-L-Cys + ADP + P_i_ (1)

γ-L-Glu-L-Cys + Gly + ATP ⇌ γ-L-Glu-L-Cys-Gly + ADP + P_i_ (2)

GSH is one of the most important low-molecular-mass thiols participating in the cellular antioxidative defense system. The intracellular concentration of GSH is in the lower mM-range, whereas the concentration of GSH in extracellular compartments is in the lower µM-range. The oxidized forms of glutathione are its symmetric disulfide GSSG and its asymmetric disulfides with high-molecular-mass thiols, including human serum albumin [5]. The intracellular GSH-to-GSSG molar ratio is generally used as a measure of cellular oxidative stress in health and disease [5].

*S*-Glutathionylation is a post-translational modification (PTM), by which GSH is formally added to the sulfhydryl (SH) group of Cys moieties of proteins. Protein *S*-glutathionylation is involved in oxidative stress; it prevents irreversible oxidation of protein thiols, and controls cell-signaling pathways [6,7]. Quantitative determination of GSH residues in proteins, i.e., of the protein *S*-glutathionylation in biological fluids and tissue, is of particular importance in numerous areas of research, including oxidative stress [8].

Inherent thermal lability and the lack of volatility in small peptides, such as GSH, represent a significant challenge for their gas chromatographic (GC) analysis. The GC-MS analysis of GSH succeeded after its derivatization to its *N,S*-carboxyethyl dimethyl esters [9,10,11]. These derivatives require electron ionization (EI), which lacks analytical sensitivity due to strong fragmentation. More recently, we reported the derivatization of GSH with 2 M HCl in CH_3_OH to its dimethyl ester and its subsequent acylation using pentafluoropropionic anhydride (PFPA) [12]. Derivatives rich in fluorine (F) atoms are more volatile than non-perfluorated derivatives and, most importantly, they are very strong electron-captors due to the highest electronegativity of F among the elements. These physicochemical features render the perfluorated derivatives best suitable for electron-capture negative-ion chemical ionization (ECNICI). This “soft” ionization method found wide application in quantitative measurements of endogenous compounds in the pM-to-nM range and possesses great potential to provide the highest currently available analytical sensitivity in GC-MS [13].

Because of the highly characteristic structural feature of GSH, that is its γ-L-glutamyl moiety, the aim of the present study was to develop a stable-isotope dilution GC-MS method for the specific measurement of γ-L-glutamyl residues. For this, we used GSH, GSSG, γ-glutamyl-cysteine (γ-Glu-Cys) and the natural GSH tripeptide analog ophthalmic acid (OPH; γ-glutamyl-α-amino-*n*-butyryl-glycine). In OPH, the Cys group of GSH is replaced by L-2-aminobutyrate. We used a two-step derivatization procedure, previously proven to be suitable for the GC-MS analysis of various classes of compounds, including amino acids and drugs [14,15,16,17,18,19]. Analysis of amino acids and their metabolites includes: 1) the preparation of their methyl esters (Me) using 2 M HCl in CH_3_OH; and 2) their subsequent acylation with pentafluoropropionic anhydride (PFPA) in ethyl acetate to prepare the pentafluoropropionyl (PFP) derivatives. Upon recognition of pyroglutamate (pGlu, 5-oxo-proline) formation, we also tested the utility of pentafluorobenzyl bromide (PFB-Br), a versatile derivatization reagent in chromatography [13], for the derivatization of pGlu. In living cells, pGlu is derived from γ-glutamyl peptides through the catalytic action of γ-glutamyl cyclotransferase (EC 2.3.2.4) [20]. This is exemplified for (5-γ-L-Glu)-L-Phe (R3).

(5-γ-L-Glu)-L-Phe ⇌ 5-oxo-proline + L-Phe(3)

## 2. Materials and Methods

### 2.1. Chemicals and Materials

GSH, GSSG, γ-Glu-Cys, Cys-Gly, 5-oxo-proline, Glu and the other amino acids used in the study, CH_3_OH, CD_3_OD (99.8 atom % ^2^H), acetone, pentafluoropropionic anhydride, 2,3,4,5,6-pentafluorobenzyl bromide, ophthalmic acid (γ-glutamyl-α-amino-*n*-butyryl-glycine) and borax (for the preparation of borate buffer) were purchased from Sigma-Aldrich (Steinheim, Germany). Hydrochloric acid (ultrapure, 37%) was from AppliChem (Darmstadt, Germany). Stock solutions were prepared in, and diluted with, deionized water, as appropriate. Glassware for GC-MS (1.8-mL autosampler vials and 0.2-mL microvials) were purchased from Macherey-Nagel (Düren, Germany).

*Safety Considerations*. PFPA is corrosive and malodorous. PFB-Br is corrosive and a lachrymator. Inhalation and contact with skin and eyes should be avoided. All work should be, and was, performed in a well-ventilated fume hood.

### 2.2. Derivatization Procedures

#### 2.2.1. Derivatization with HCl/Methanol and Pentafluoropropionic Anhydride

A two-step derivatization reaction was used. The first derivatization reaction was separate esterification of GSH, GSSG, ophthalmic acid, 5-oxo-proline, proline, hydroxy-proline, glutamate, and other amino acids. For this, solid residues were dissolved in 200-μL aliquots of 2 M HCl/CH_3_OH or 2 M HCl/CD_3_OD and the samples were heated in tightly closed glass vials for 60 min at 80 °C [14]. Analytes derivatized with 2 M HCl/CD_3_OD served as internal standards. This procedure results in the formation of the unlabeled and deuterium-labeled methyl esters (d_0_Me and d_3_Me, respectively) of the mono- and di-carboxylic amino acids and peptides. After cooling to room temperature, the solvents were evaporated to dryness by a stream of nitrogen gas. The second derivatization reaction was then performed by dissolving the methyl esters in 100-μL aliquots of a solution of PFPA in ethyl acetate (1:4, *v/v*), and by heating the tightly sealed glass vials for 30 min at 65 °C. After cooling to room temperature, the solvent and reagents were evaporated to dryness under a stream of nitrogen gas. The residues were then reconstituted with 200-μL aliquots of 0.4 M borate buffer, pH 8.5, and the samples were immediately mixed by vortexing for 60 s with toluene (1000 µL). Aliquots (100–800 μL, as appropriate) of the upper organic layer were subsequently taken and transferred into autosampler glass vials for GC-MS analysis. The lower aqueous phase was discarded.

#### 2.2.2. Derivatization with Pentafluorobenzyl Bromide

Synthetic pyroglutamate (10 nmol from a freshly prepared aqueous solution) was diluted with acetone (500 µL) and PFB-Br (10 µL of a 30 vol% solution in acetonitrile) was added. The sample was sealed and heated for 60 min at 50 °C. After cooling to room temperature, acetone was removed by means of a nitrogen gas stream. The residue was extracted with ethyl acetate (1000 µL) by vortexing for 1 min. After centrifugation (5 min, 3225× *g*), a 900-µL aliquot of the upper organic phase was decanted and dried over anhydrous Na_2_SO_4_. Finally, 750-µL of the supernatant was transferred into autosampler vials for GC-MS analysis.

### 2.3. GC-MS Conditions

GC-MS analyses were performed on a ThermoElectron ISQ quadrupole mass spectrometer connected directly to a Thermo-Electron Focus gas chromatograph and to an autosampler AS 3000 (ThermoElectron, Dreieich, Germany). A fused-silica capillary column Optima-17 (15 m × 0.25 mm internal diameter, 0.25-µm film thickness) from Macherey-Nagel (Düren, Germany) was used. Aliquots (1 µL) of toluene or ethyl acetate extracts were injected in the splitless mode. Two oven temperature programs were used with helium (1 mL/min) as the carrier gas. In temperature program (TP) #1, the oven was held for 1 min at 70 °C, then increased to 280 °C at a rate of 30 °C/min, and finally to 300 °C at a rate of 10 °C/min. In temperature program TP#2, the oven was held for 0.5 min at 40 °C, then increased to 210 °C at a rate of 15 °C/min, and finally to 320 °C at a rate of 35 °C/min. Interface, injector and ion-source were kept constant at 260 °C, 200 °C and 250 °C, respectively. The electron energy and emission current were set to 70 eV and 120 µA, respectively. ECNICI was performed with methane (1 mL/min) as the reagent gas.

GC-MS spectra were obtained in the scanning mode in the mass-to-charge (*m*/*z*) range of 100 to 800 or 100 to 1000, at a rate of 1 scan/s. Quantitative analyses were performed in the selected-ion monitoring (SIM) mode. Peak area values were calculated automatically by the GC-MS software (Xcalibur and Quan Browser; ThermoElectron, Dreieich, Germany). Statistical analyses were performed, and graphs were prepared by GraphPad Prism 7 (San Diego, CA, USA). Chemical structures were drawn by using ChemDrawProfessional 15.0 (Perkin Elmer, Germany).

## 3. Results

### 3.1. Formation of Pyroglutamate from GSH

**Figure 1:**
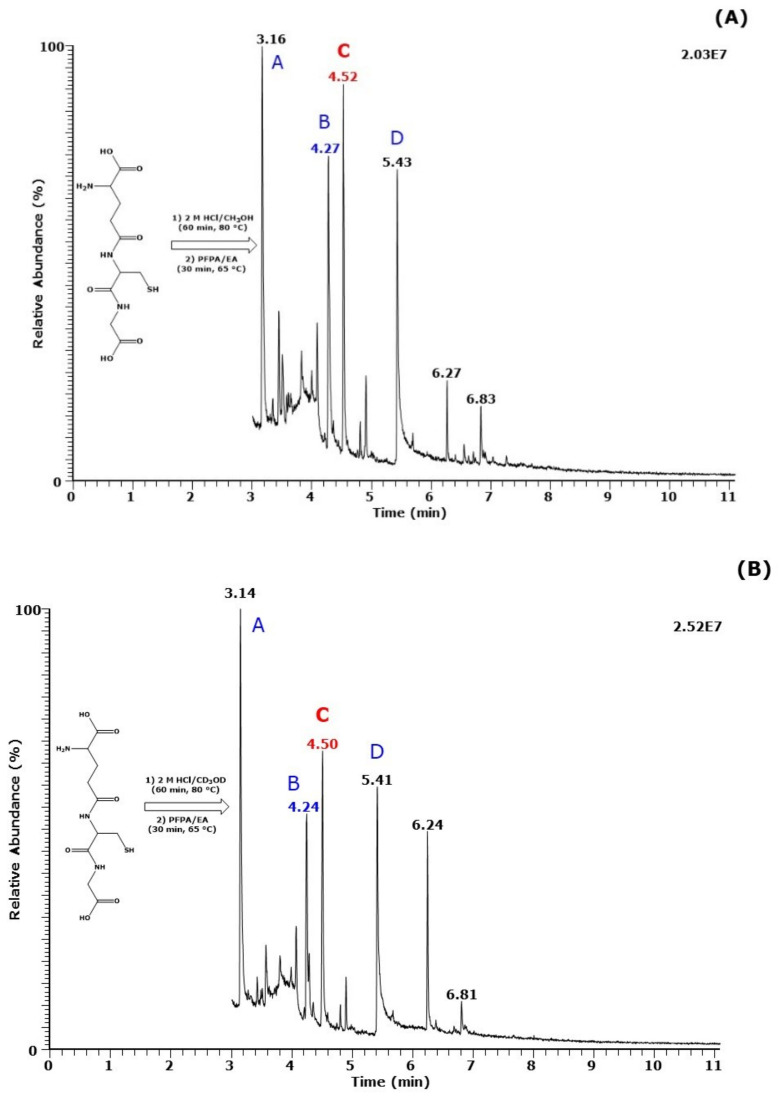
Total ion current GC-MS chromatograms in the ECNICI mode from separate analyses of glutathione (GSH, each 10 nmol) after its esterification (60 min, 80 °C) in 2 M HCl/CH_3_OH (**A**) or 2 M HCl/CD_3_OD (**B**) and subsequent acylation with PFPA (30 min, 65 °C). Oven temperature program TP#1 was used. Inserts indicate the chemical structure of GSH and the derivatization conditions. The quadrupole was scanned in the mass-to-charge (*m*/*z*) *m*/*z*-range 100–1000 (1 scan/s). The mass spectra of the peaks A, B, C and D are shown in Figure 2.

**Figure 2:**
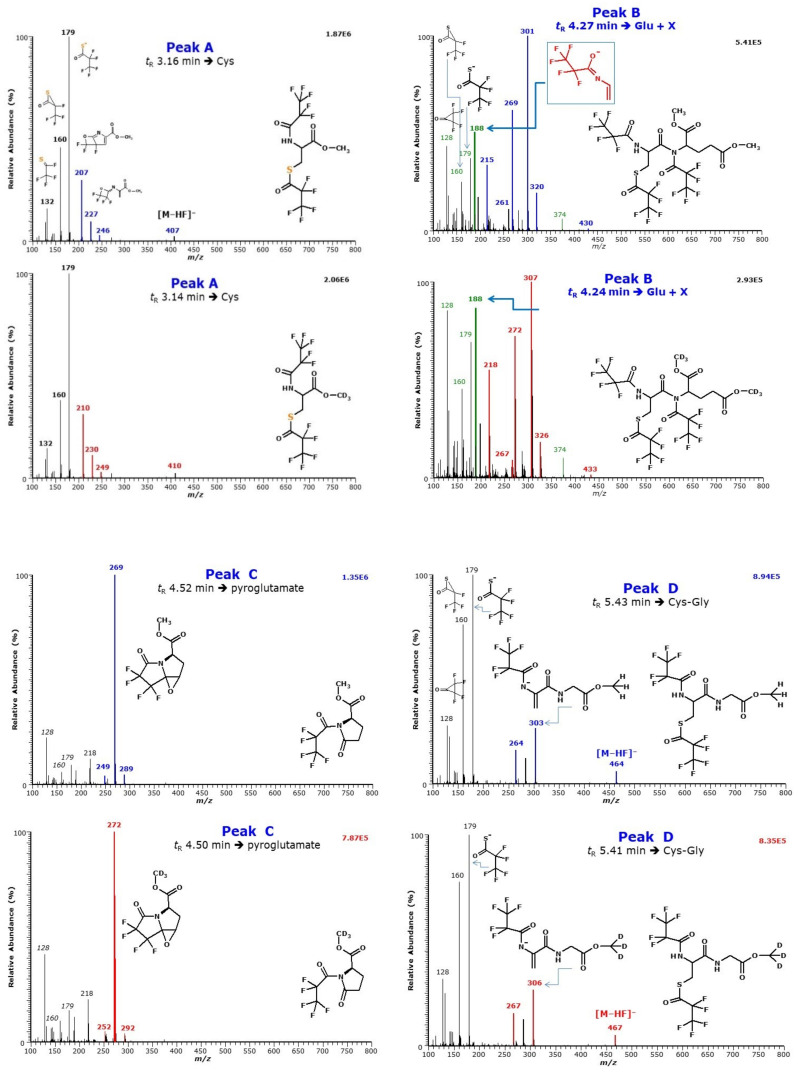
GC-MS spectra in the ECNICI mode of the peaks A, B, C and D, obtained from separate analysis of GSH (each 10 nmol) after its esterification (60 min, 80 °C) in 2 M HCl/CH_3_OH or 2 M HCl/CD_3_OD and subsequent acylation with PFPA (30 min, 65 °C). Oven temperature program TP#1 was used. The quadrupole was scanned in the *m*/*z*-range 100–1000 (1 scan/s). Inserts indicate the proposed structures for the derivatives and the mass fragments. The B peaks contain Glu and Glu-Cys (X). Peaks A, B, C and D refer to Figure 1.

GC-MS analysis of the GSH that was first esterified with 2 M HCl/CH_3_OH (Figure 1A) or 2 M HCl/CD_3_OD (Figure 1B), and then acylated with PFPA, revealed similar chromatograms in the ECNICI mode. They each contained four major GC peaks (Figure 1). The GC-MS peaks A, B, C and D of the trideuteromethyl ester (d_3_Me) pentafluoropropionic (PFP) derivatives (d_3_Me-PFP) (Figure 1B) eluted a few seconds before the corresponding non-deuterated methyl ester PFP derivatives (d_0_Me-PFP) (Figure 1A), indicating the presence of deuterium atoms in their molecules. Analysis of these samples in the positive-ion chemical ionization (PICI) mode revealed only the GC-MS C peaks (data not shown). PICI was not used further in the study.

A peaks (retention time, 3.16/3.14 min) were identified as the Cys derivatives (Figure 2A) by using synthetic Cys (not shown). The mass fragments *m*/*z* 132, *m*/*z* 160 and *m*/*z* 179 are common to Cys-d_0_Me-PFP and Cys-d_3_Me-PFP and are likely to contain the sulfur (S) atom of Cys. The corresponding mass fragments *m*/*z* 207, *m*/*z* 227, *m*/*z* 246 and *m*/*z* 407 (Figure 2A) and *m*/*z* 210, *m*/*z* 230, *m*/*z* 249 and *m*/*z* 410 (Figure 2B) each differ by 3 Da indicating the presence of esterified carboxylic groups d_0_Me and d_3_Me, respectively. These results indicate that under the used derivatization procedures, GSH is hydrolyzed to form Cys, which is thereby converted to its methyl ester *N,S*-PFP derivatives (i.e., Cys-d_0_Me- (PFP)_2_ and Cys-d_3_Me-(PFP)_2_).

The mass spectra of the GC-MS B peaks (retention time, 4.27/4.24 min) contained non-differing mass fragments at *m*/*z* 128, *m*/*z* 160, *m*/*z* 179, *m*/*z* 188, and *m*/*z* 374, and matched mass fragments differing by 3 Da (one d_3_Me group), i.e., *m*/*z* 215 and *m*/*z* 218, *m*/*z* 269 and *m*/*z* 272, and *m*/*z* 430 and *m*/*z* 433, as well as matched mass fragments differing by 6 Da (two d_3_Me groups), i.e., *m*/*z* 261 and *m*/*z* 267, *m*/*z* 301 and *m*/*z* 307, and *m*/*z* 320 and *m*/*z* 326 (Figure 2B). The latter mass fragments were also found in the GC-MS spectra of derivatized synthetic Glu [14], suggesting that Glu is present in the GC-MS B peaks. The appearance of additional mass fragments in the mass spectrum of the GC-MS B peaks indicates the co-elution of an additional component. The mass fragments *m*/*z* 261 and *m*/*z* 264, *m*/*z* 269 and *m*/*z* 272, and *m*/*z* 301 and *m*/*z* 307 were the most intense ions in the GC-MS mass spectra of the unlabeled and deuterium-labeled methyl ester of synthetic γ-Glu-Cys (data not shown). These observations suggest that B peaks also contain γ-Glu-Cys (labeled as X in Figure 2B), generated from GSH during the derivatization procedures.

The mass spectra of the GC-MS peaks C (retention time, 4.52/4.50 min) contained mass fragments that differed by 3 Da (one d_3_Me group), but did not contain mass fragments differing by 6 Da (two d_3_Me groups) (Figure 2. These observations suggest that the GC-MS C peaks are due to a mono-carboxylic amino acid derivative. Both the mass spectra and the retention times of the derivatives of proline (Pro) and hydroxyproline (Hyp) [14] differed from those of the C peaks (Figure 2). Thus, neither Pro nor Hyp are formed during the derivatization of GSH.

The mass spectra of the GC-MS D peaks (retention time, 5.43/5.41 min) contained intense mass fragments that did not differ among themselves (*m*/*z* 128, *m*/*z* 160, *m*/*z* 179), but did contain matched mass fragments differing by 3 Da (*m*/*z* 264, *m*/*z* 267, *m*/*z* 303, *m*/*z* 306, *m*/*z* 464, *m*/*z* 467) due to one d_3_Me group (Figure 2). Both the mass spectra and the retention times of the derivatives of synthetic Cys-Gly (data not shown) were very similar to those of the GC-MS D peaks, suggesting GSH hydrolysis during the esterification procedure to form Me-PFP derivatives of the dipeptide Cys-Gly.

**Figure 3:**
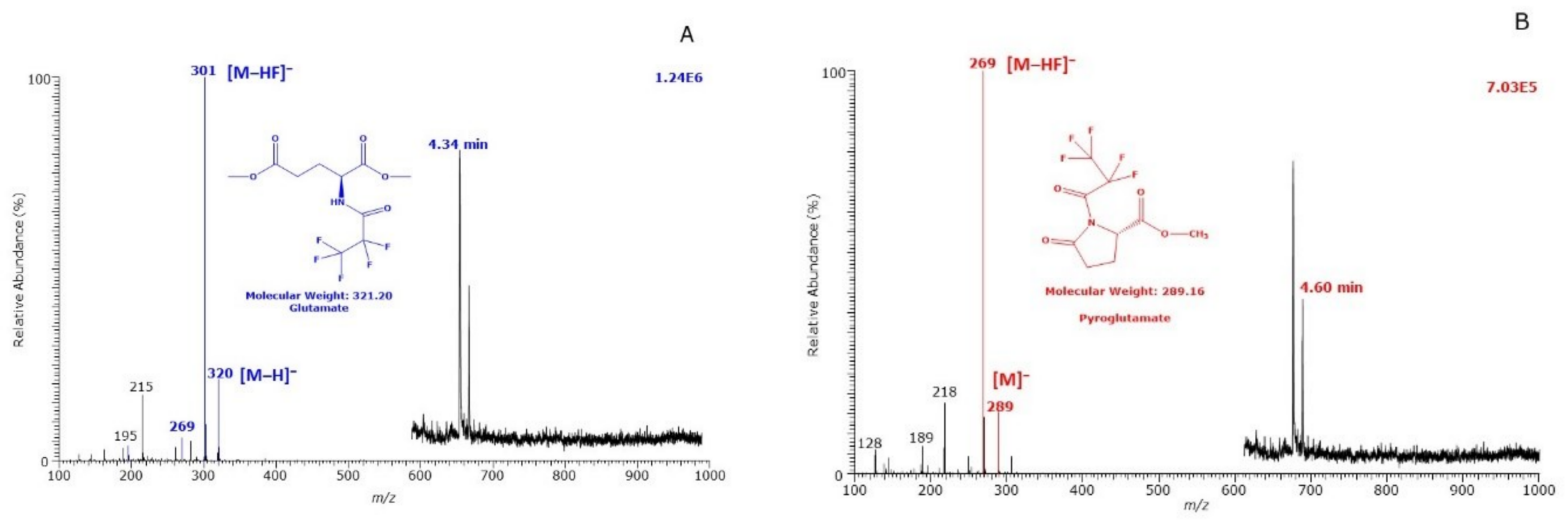
Mass spectra in the ECNICI mode and chromatograms (inserted) of the two GC-MS peaks, with the retention times 4.34 and 4.60 min, obtained from the derivatization of synthetic pyroglutamate (10 nmol) after its esterification (20 min, 80 °C) in 2 M HCl/CH_3_OH and subsequent acylation with PFPA (30 min, 65 °C). The GC-MS peak eluting at 4.34 min (**A**) was identified as the glutamate (Glu) derivative. The GC-MS peak with the retention time 4.60 min (**B**) is most likely due to the Me-PFP derivative of pyroglutamate (pGlu). Oven temperature program TP#1 was used. The quadrupole was scanned in the *m*/*z*-range 100–1000 (1 scan/s). Inserts indicate the proposed structures for selected mass fragments. M, molecular mass of the derivative.

In theory, pyroglutamate (pGlu; 5-oxo-proline or 5-keto-proline) can also be formed during the derivatization of GSH. Derivatization of synthetic pGlu with 2 M HCl/MeOH (20 min, 80 °C), and then with PFPA (30 min, 65 °C), resulted in the formation of two GC-MS peaks (Figure 3). The major peak, with a retention time of 4.34 min, was identified as Glu (Figure 3A). Clearly, authentic pGlu is converted in part to Glu under the derivatization conditions. The mass spectrum of the smaller GC-MS peak eluting behind the Glu peak, with a retention time of 4.60 min (Figure 3B), was very similar to the mass spectrum of peak C (Figure 2). The most characteristic mass fragments were *m*/*z* 289 due to [M]^−^ (molecular anion) and *m*/*z* 269 due to [M–HF]^−^ (loss of one HF molecule, 20 Da, from the molecular anion) in the mass spectrum of pGlu derivatized with 2 M HCl/MeOH (Figure 3B). The most likely candidate for the GC-MS C peak is pyroglutamate (pGlu). Figure 3B suggests that the secondary amine group of pGlu is acylated with PFPA.

Neither synthetic glutamine (Gln) nor synthetic Glu produced the C peaks upon esterification and *N*-acylation under the same conditions. Therefore, pGlu seems to have been formed intra-molecularly from GSH during the first derivatization step, but not during the ionization in the ion-source of the GC-MS apparatus. It is worth mentioning that in LC-MS/MS, Gln and Glu undergo in-source conversion/cyclization to pGlu [21].

### 3.2. Formation of Pyroglutamate from Ophthalmic Acid

**Figure 4:**
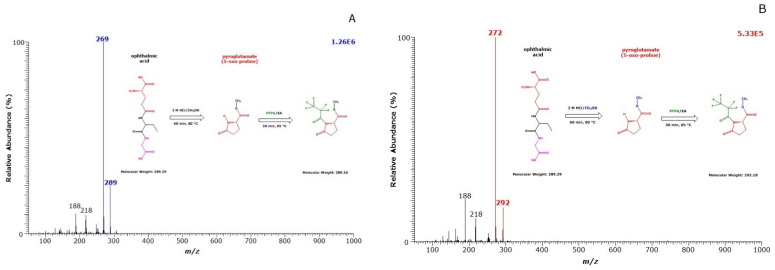
ECNICI mass spectra of the GC-MS peaks obtained from the derivatization of synthetic OPH (each 10 nmol) after its esterification (20 min, 80 °C) in 2 M HCl/CH_3_OH (A) or in 2 M HCl/CD_3_OD, and subsequent acylation with PFPA in ethyl acetate (EA) (30 min, 65 °C). The retention times of the derivatives were 8.43 min (**A**) and 8.41 min (**B**), using TP#2. The quadrupole was scanned in the *m*/*z*-range 80–1000 (1 scan/s). Inserts indicate the chemical structure of OPH, the derivatization conditions and the proposed structures of the methyl ester and pentafluoropropionyl derivatives.

Ophthalmic acid (γ-glutamyl-α-amino-n-butyryl-glycine; OPH) is a tripeptide analogue of GSH. GC-MS analysis of OPH first esterified with 2 M HCl/CH_3_OH or 2 M HCl/CD_3_OD, and subsequently acylated with PFPA, revealed very similar chromatograms in the ECNICI mode (not shown). The mass spectrum of the GC-MS peak eluting at 8.43 min, using TP#2 (Figure 4), was virtually identical to the mass spectrum of synthetic pGlu (Figure 3B). The most intense ions were *m*/*z* 289 and *m*/*z* 269 for OPH derivatized with unlabeled methanol (Figure 4A). The most intense ions in the mass spectrum of the GC-MS peak eluting at 8.39 min, obtained from the derivatization of OPH with deuterium-labeled methanol, were *m*/*z* 292 and *m*/*z* 272 (Figure 4B). These results strongly suggest that the γ-glutamyl tripeptide OPH is also converted to the methyl ester of pGlu during the esterification procedure.

### 3.3. Kinetics of Pyroglutamate Formation from GSH

**Figure 5:**
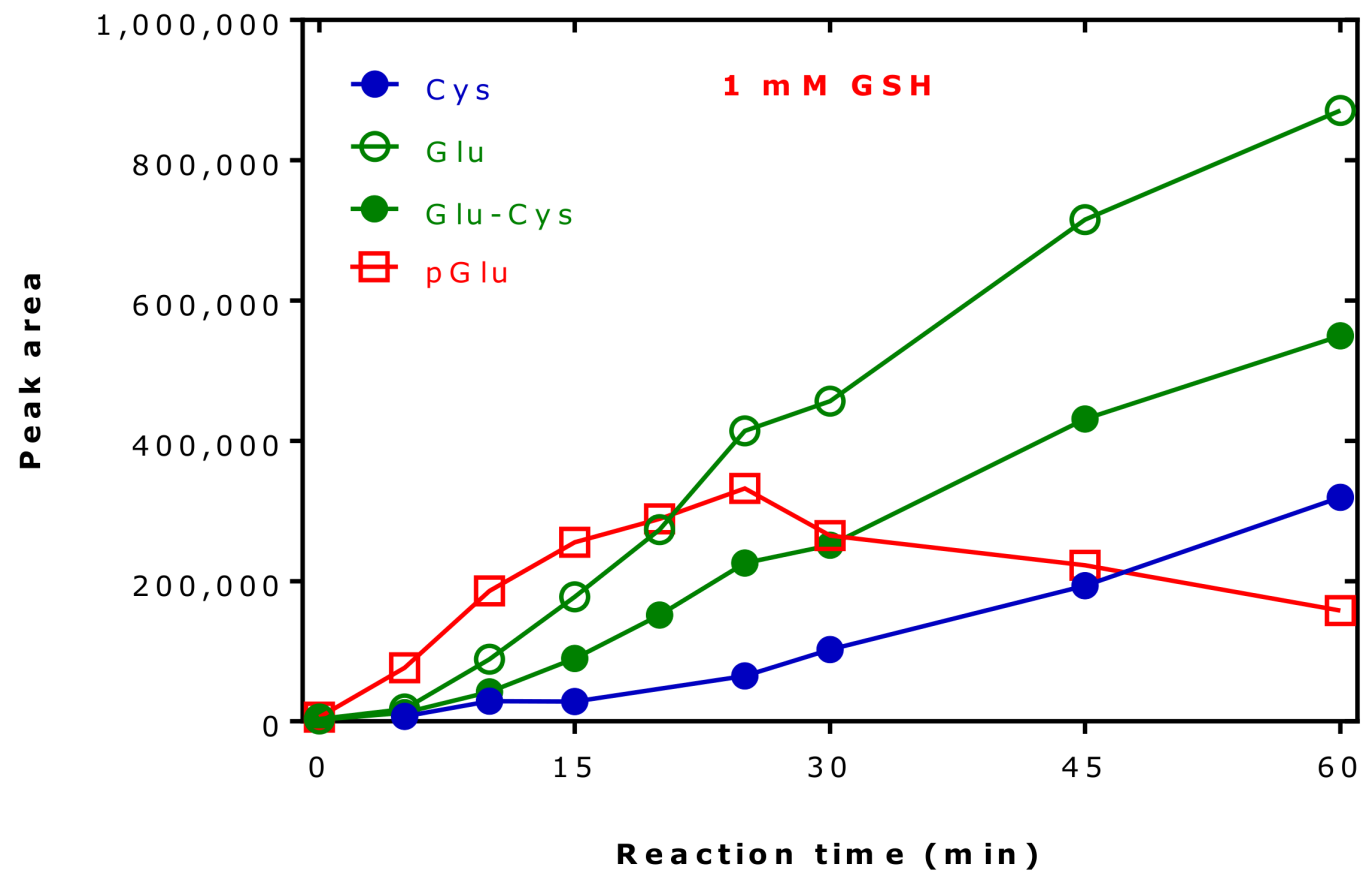
Peak area values of the GC-MS peaks of Cys (Peak A, *m*/*z* 207), of Glu/Glu-Cys (Peak B and C, *m*/*z* 269 and *m*/*z* 301) or pGlu (Peak C, *m*/*z* 269) from the esterification of GSH (1000 µM) at 80 °C for the indicated reaction times. Subsequent acylation was performed with PFPA (30 min, 65 °C). Peaks A, B and C refer to Figure 1.

In the experiments described above, we used the conditions of the standard esterification procedure previously developed and used for the quantification of individual amino acids [13], i.e., heating at 80 °C for 60 min in 2 M HCl/methanol. As pGlu is formed from GSH, we investigated the effect of the esterification time on the formation of Cys, Glu/Glu-Cys and pGlu from GSH (1 mM) during its derivatization at 80 °C. Figure 5 shows steadily increasing peak areas for Cys and Glu/Glu-Cys with increasing reaction time, whereas the peak area of pGlu reaches a maximum after 25 min of reaction. Clearly, pGlu is an intermediate in the hydrolysis of GSH under the conditions of the esterification procedure. It seems that pGlu is the first reaction product, which further reacts to form the dimethyl ester of Glu, a dicarboxylic amino acid. The intermediate formation of pGlu is supported by the finding that esterification of synthetic pGlu results in abundant formation of Glu, as described above (Figure 4). For the measurement of GSH in GSH-rich media by GC-MS as pGlu, an esterification time of about 20 to 30 min would be optimum (Figure 5).

### 3.4. Quantitative GC-MS Analysis of GSH-Derived pGlu as Me-PFP Derivative

We prepared two standard curves (A and B) by separate esterification with 2 M HCl/CH_3_OH of eight samples containing 0, 0.25, 0.5, 1, 2.5, 5, 7.5 and 10 nmol of GSH. After the esterification, newly prepared hexadeuterated GSH (d_6_-GSH) [12] was added to each sample for use as an internal standard (1 nmol for standard curve A, 10 nmol for standard curve B), and the combined samples were derivatized with PFPA. Quantification was performed by SIM: the ions *m*/*z* 301 and *m*/*z* 307 for peak B (presumably Glu and Glu-Cys, i.e., Glu/Glu-Cys), and the ions *m*/*z* 269 and *m*/*z* 272 for peak C, i.e., pGlu.

**Figure 6:**
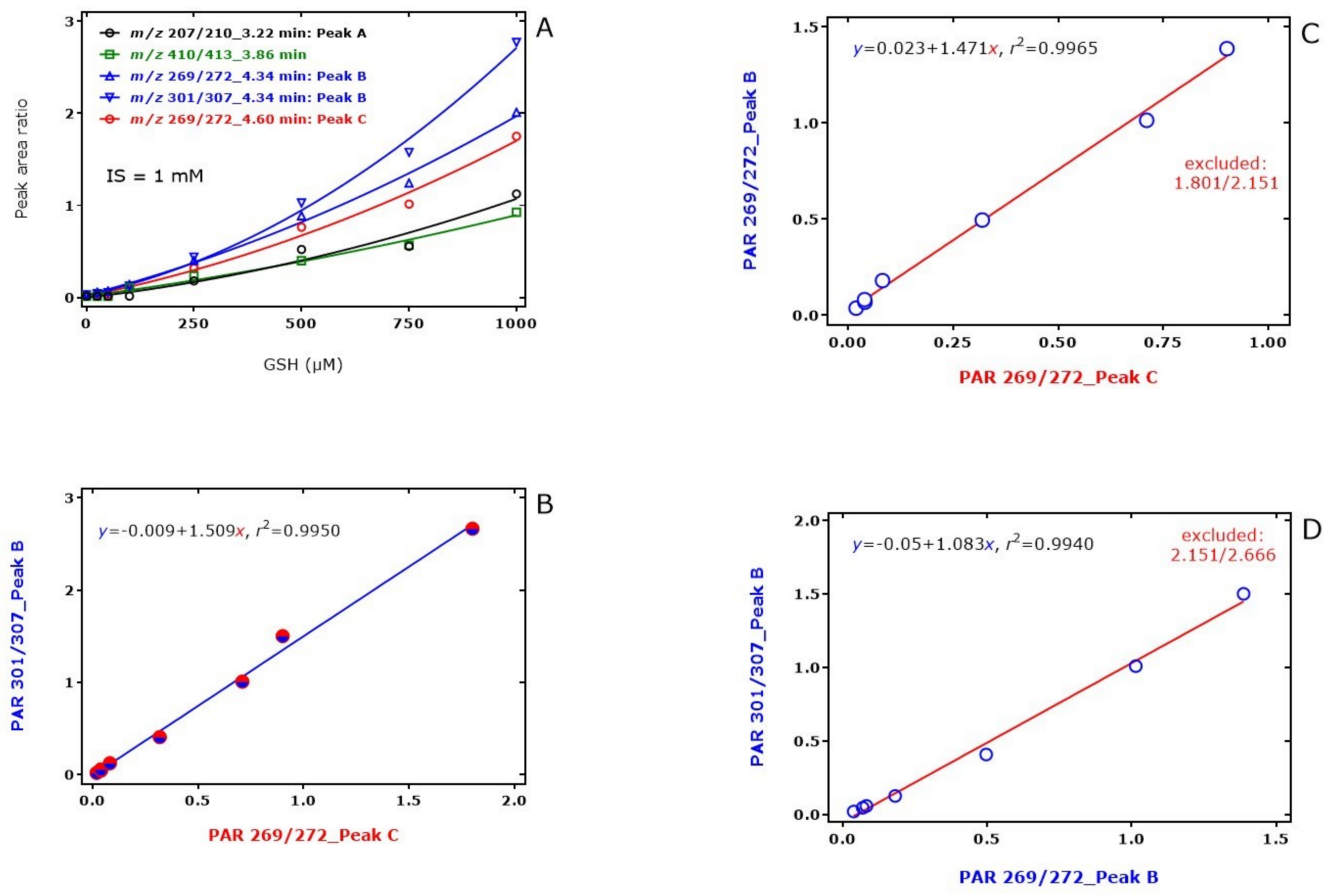
(**A**) Peak area ratio values for the GC-MS peaks A, B and C, formed from derivatized GSH (0–1000 µM) using 1 mM d_6_-GSH as internal standard (IS). (**B**) Linear regression analysis between the peak area ratio (PAR) of *m*/*z* 301 to *m*/*z* 307 for peak B, and of the PAR *m*/*z* 269 to *m*/*z* 272 for peak C. (**C**) Linear regression analysis between the PAR of *m*/*z* 269 to *m*/*z* 272 for peak B, and the PAR of *m*/*z* 269 to *m*/*z* 272 for peak C. (**D**) Linear regression analysis between PAR of *m*/*z* 301 to *m*/*z* 307 for peak B, and the PAR of *m*/*z* 269 to *m*/*z* 272 for peak B with the concentration of glutathione (GSH) in the range 0–1000 µM. Note the two excluded values in (**C**,**D**). Oven temperature program TP#1 was used. Peaks A, B and C refer to Figure 1.

For the standard curve A, we observed a non-linear increase in the peak area ratio (PAR) of peak B (PAR_B_), but a linear increase in the PAR of peak C (PAR_C_) (*y*) with increasing GSH concentration (*x*): *y* = −0.342 + 0.013*x*, *r*^2^ = 0.9908 (Figure 6A). The PAR_B_-to-PAR_C_ ratio was variable (2.32 ± 0.42; relative standard deviation (RSD), 18%) due to the stronger quadratic increase in peak B (Glu/Glu-Cys). For the standard curve B, we observed non-linear increases in PAR_B_ and PAR_C_ with increasing GSH concentration (Figure 6B). Best fit between PAR_B_ or PAR_C_ and GSH concentration was obtained by second order polynomial function (*r*^2^ = 0.9989 and *r*^2^ = 0.9993, respectively). PAR_B_ and PAR_C_ correlated closely with each other (*r* = 1.000), and the PAR_B_-to-PAR_C_ molar ratio was less variable (1.42 ± 0.17; RSD, 12%) over the entire GSH concentration range.

**Figure 7:**
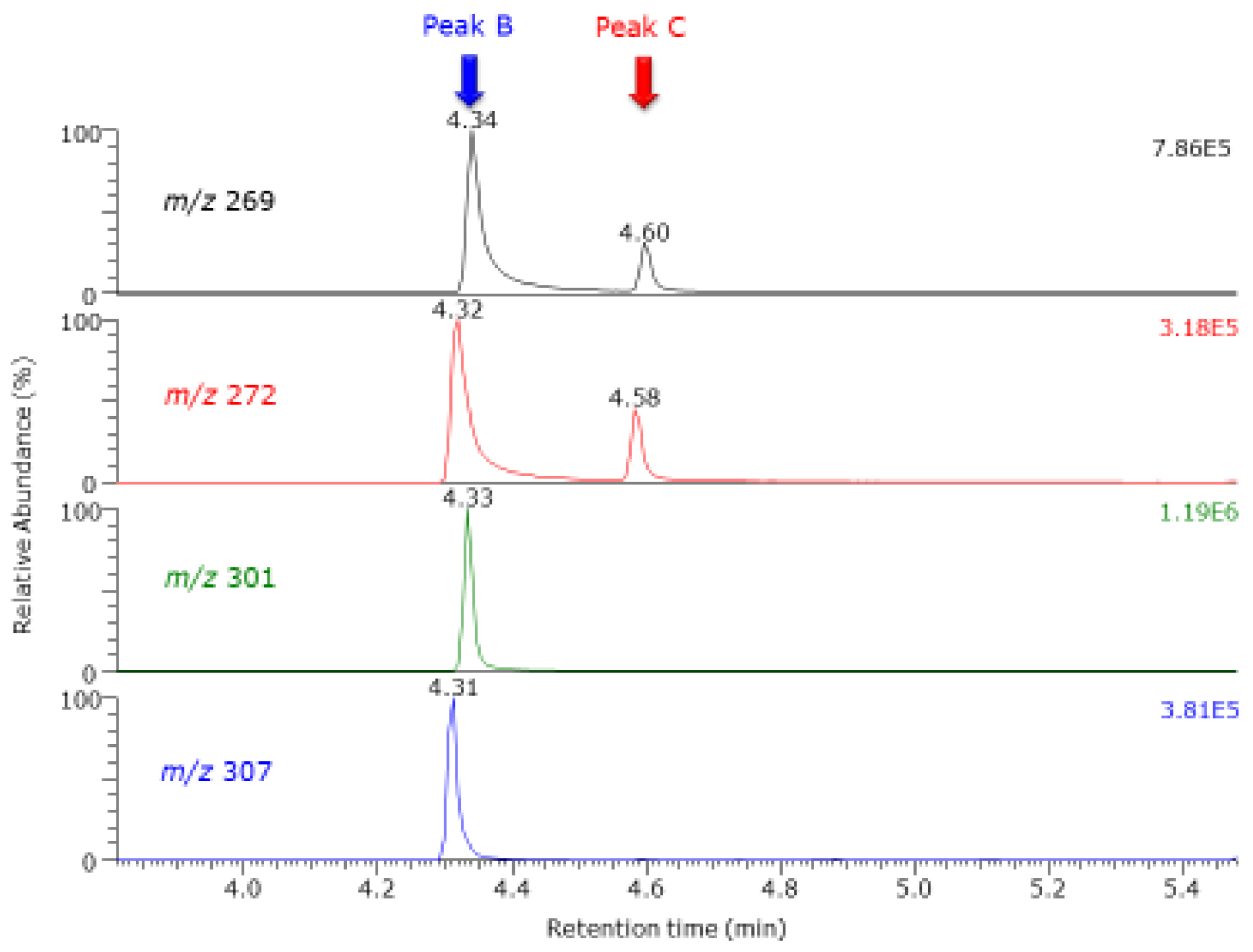
GC-MS chromatograms from the measurement of GSH (1 mM) with deuterium-labeled GSH (1 mM) as the internal standard after esterification and acylation. SIM of *m*/*z* 269, *m*/*z* 272, *m*/*z* 301 and *m*/*z* 307 was performed. Peak C eluting at 4.58/4.60 min with *m*/*z* 269 and *m*/*z* 272 is considered to be pyroglutamate from decomposed/rearranged unlabeled and deuterium-labeled GSH. Oven temperature program TP#1 was used. Peaks B and C refer to Figure 1.

A representative GC-MS chromatogram from the quantitative analysis of aqueous GSH, as Glu/Glu-Cys (peak B) and pGlu (peak C), is shown in Figure 7. The derivatives of unlabeled and deuterium-labeled pGlu with *m*/*z* 269 and *m*/*z* 272 eluted at 4.60 min and 4.58 min (peak C), respectively. Peak B seems to be heterogeneous. One component is Glu, of which the derivatives elute as sharp peaks at 4.31 min with *m*/*z* 307 for the deuterium-labeled Glu, and at 4.33 min with *m*/*z* 301 for unlabeled Glu. The second component is likely to be the dipeptide Glu-Cys, which elutes as a broad slightly tailing peak at 4.34 min with *m*/*z* 269 for labeled Glu-Cys, and at 4.32 min with *m*/*z* 272 for deuterium-labeled Glu-Cys. The linear relationships in Figure 6B–D suggest constant stoichiometric formation of the GSH-derived reaction products.

### 3.5. Quantitative GC-MS Analysis of GSSG-Derived pGlu as Me-PFP Derivative

**Figure 8:**
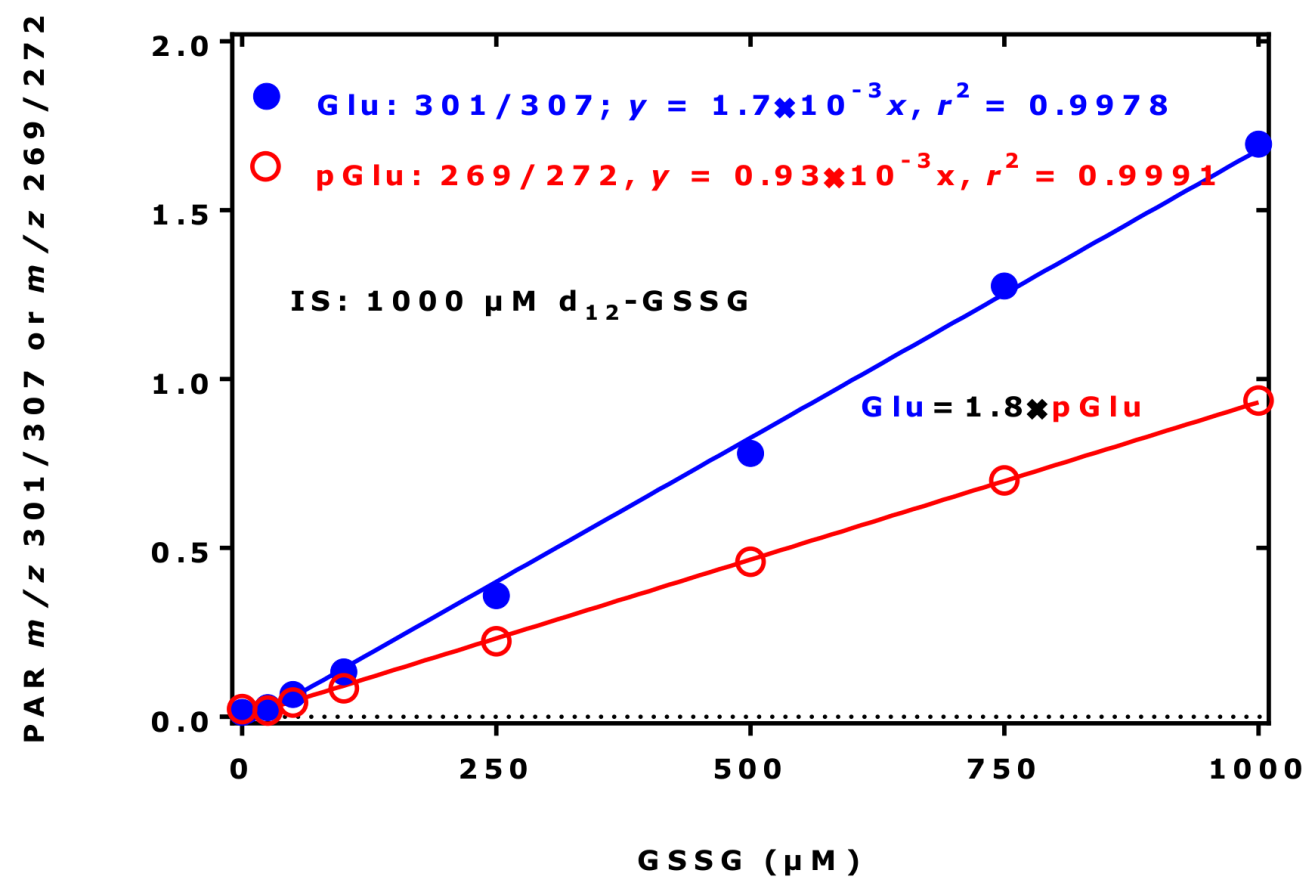
Linear relationship of the peak area ratio (PAR) of *m*/*z* 269 to *m*/*z* 272 for pGlu (Peak C) and of the PAR of *m*/*z* 301 to *m*/*z* 307 for Glu (Peak B), and the concentration of GSSG (range, 0 to 1000 µM), using d_12_-GSSG as internal standard at 1000 µM. Standard esterification (60 min, 80 °C) and acylation (30 min, 65 °C) were performed.

We prepared standard curves for aqueous GSSG, as described above for GSH. Under the same conditions for derivatization and GC-MS analysis, we observed highly linear relationships between the PAR_B_ (*m*/*z* 301 to *m*/*z* 307) for Glu/Glu-Cys or PAR_C_ (*m*/*z* 269 to *m*/*z* 272) for pGlu, and the concentration of derivatized GSSG (range, 0 to 1000 µM; 0–10 nmol), using in situ [12] prepared deuterium-labeled GSSG (d_12_-GSSG, 1000 µM; 10 nmol) as the internal standard (Figure 8). The regression equations were *y* = 1.7 × 10^−3^*x* (*r*^2^ = 0.9978) for Glu/Glu-Cys and *y* = 0.93 × 10^−3^*x* (*r*^2^ = 0.9991) for pGlu. Linear regression analysis between PAR_B_ and PAR_C_ resulted in the regression equation *y* = 1.8 × *x* (*r*^2^ = 0.999), indicating a mean Glu/Glu-Cys-to-pGlu stoichiometry of about 2:1. Thus, under the same derivatization conditions, GSSG is also converted to Glu/Glu-Cys (peak B) and pGlu (peak C) in a concentration-dependent manner.

### 3.6. GC-MS Discrimination between GSH and GSSG as Me-PFP Derivatives

**Figure 9:**
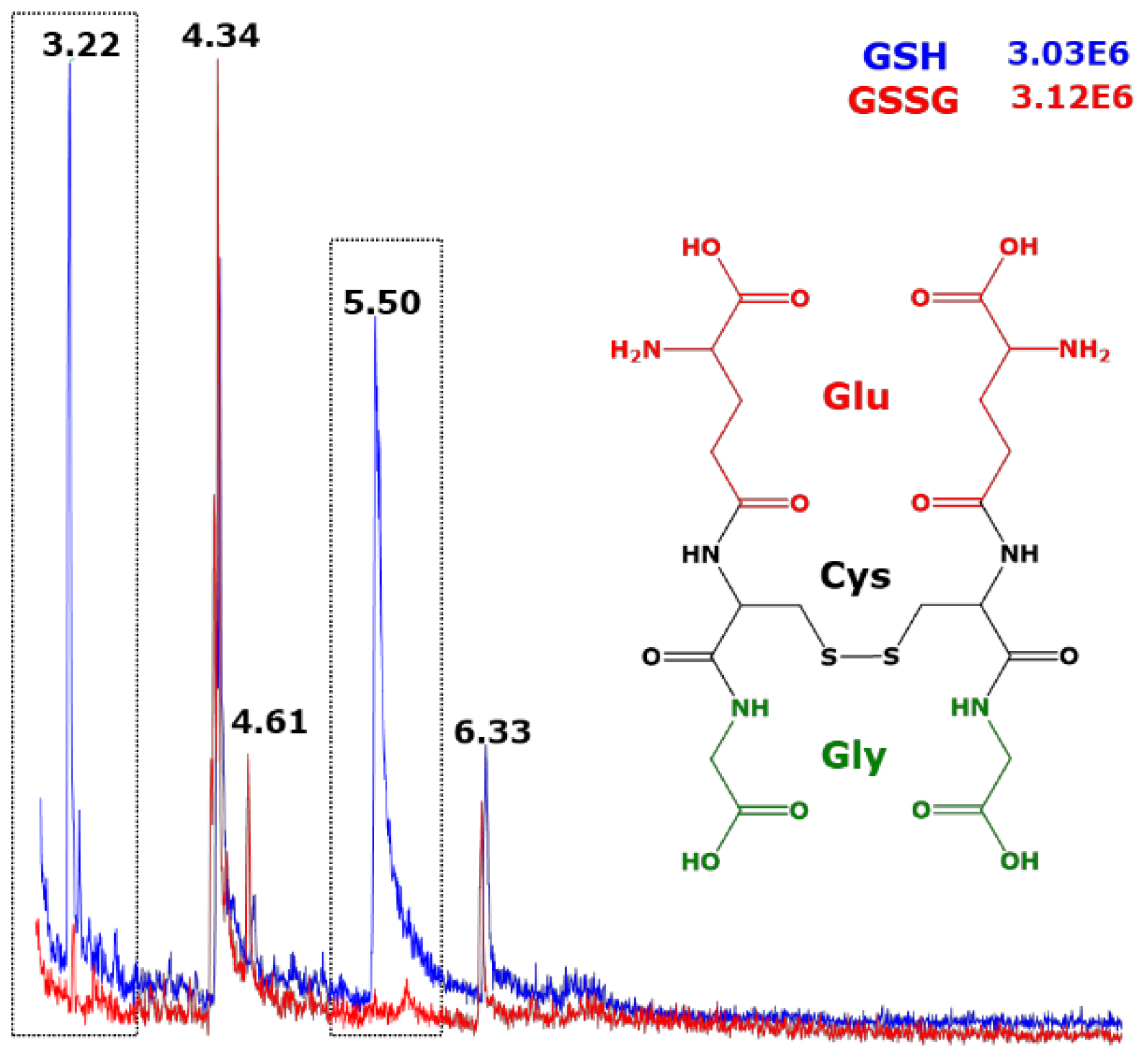
Overlaid total ion current GC-MS chromatograms in the ECNICI mode from separate analyses of synthetic reduced glutathione (GSH, blue colored) and synthetic glutathione disulfide (GSSG, red colored) (each 10 nmol) after esterification (60 min, 80 °C) in 2 M HCl/CH_3_OH, and subsequent acylation with PFPA (30 min, 65 °C). The quadrupole was scanned in the *m*/*z*-range 100–800 (1 scan/s). Oven temperature program TP#1 was used. Insert indicates the chemical structure of GSSG. Color within the GSSG structure was used for the amino acid components of GSSG. The numbers 3.03E6 (i.e., 3.03 × 10^6^) and 3.12E6 (i.e., 3.12 × 10^6^) indicate the total ion current in arbitrary units in the chromatograms of GSH and GSSG, respectively.

**Figure 10:**
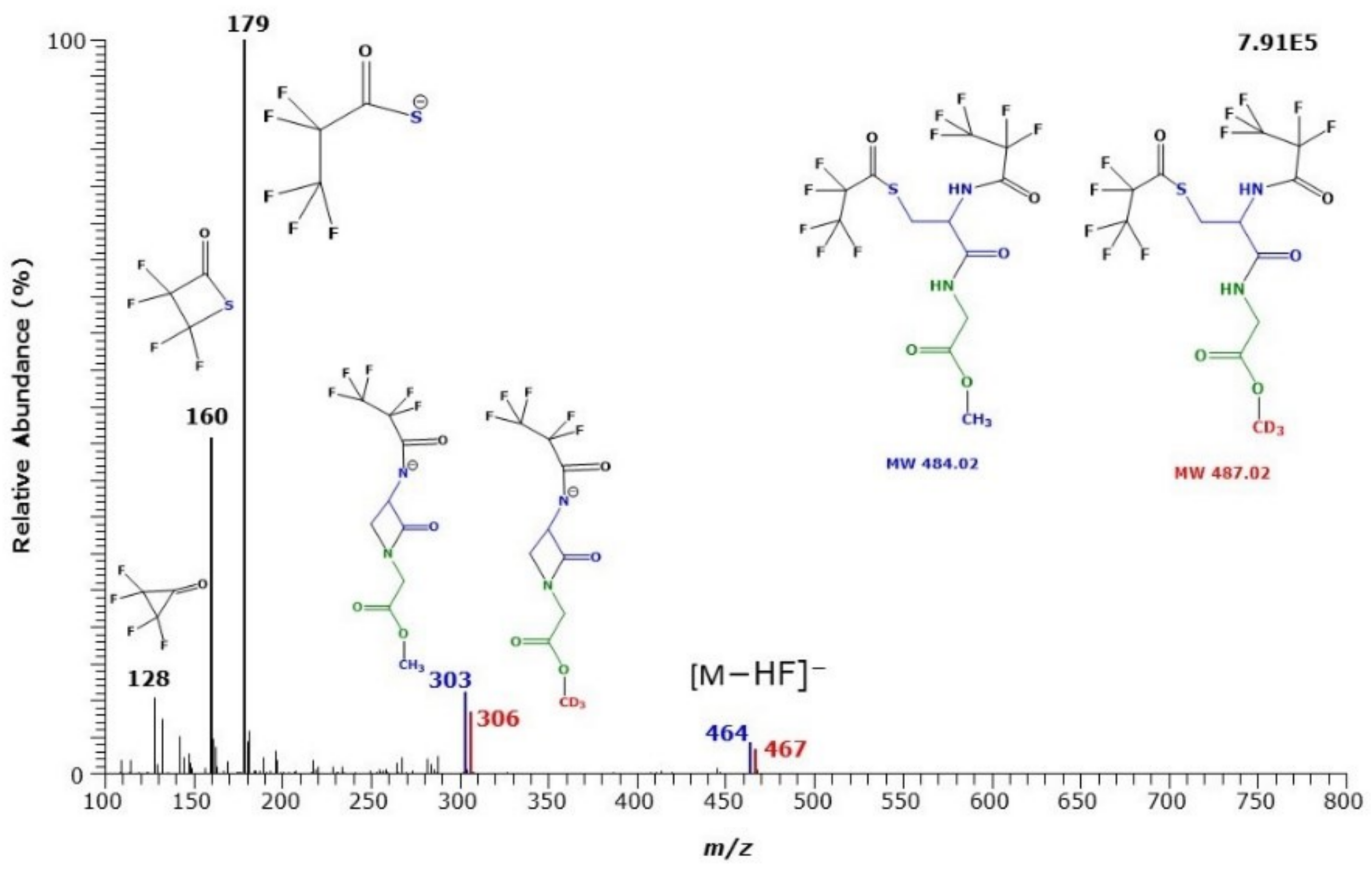
ECNICI mass spectra: GC-MS peaks with retention time of 5.50 min (TP#1), obtained from the separate derivatization of two 10 nmol aliquots of synthetic glutathione disulfide (GSSG) with 2 M HCl/CH_3_OH or 2 M HCl/CD_3_OD, and subsequent combined acylation with PFPA (30 min, 65 °C). The quadrupole was scanned in the *m*/*z*-range 100–800 (1 scan/s). Inserts indicate the proposed structures of the derivatives and the mass fragments. M, molecular mass; MW, molecular weight.

Figure 9 shows overlaid GC-MS chromatograms from the separate two-step derivatization of synthetic GSH and GSSG. As reported above, derivatization of GSH produced four peaks previously denoted as A, B, C and D (see Figure 1). Derivatization of GSSG produced two peaks. The peak with a retention time of 4.63 min corresponds to pGlu. The GC-MS spectra of the peaks with a retention time of 5.50 min are virtually identical to those of the derivatives of synthetic Cys-Gly (data not shown). The GSH-derived peak at 5.50 min is most likely due to the Cys-Gly derivative and seems to be exclusively produced from the derivatization of GSH (Figure 10). Thus, esterified and acylated GSH and GSSG both form pGlu, but can be discriminated by GC-MS after esterification and acylation, as described in this work. The ions *m*/*z* 179 and *m*/*z* 160 are common to labeled and unlabeled GSH and indicative of its Cys moiety.

### 3.7. GC-MS Analysis of pGlu as Pentafluorobenzyl Ester

**Figure 11:**
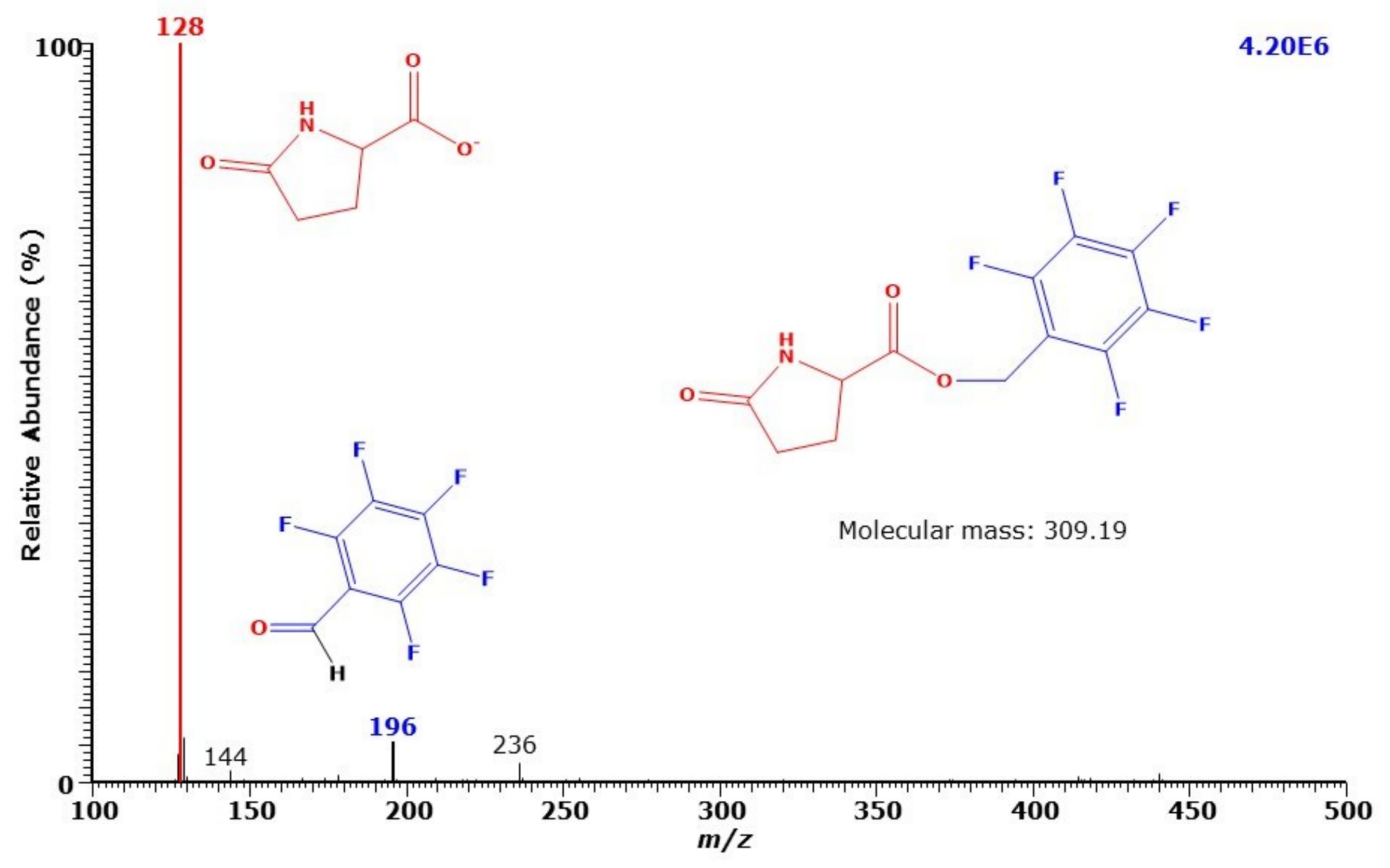
Mass spectrum in the ECNICI mode of the GC-MS peak with the retention time of 7.18 min (TP#1) obtained from the derivatization of synthetic pyroglutamate (5-oxo-proline, 10 nmol) with PFB-Br (60 min, 50 °C). The quadrupole was scanned in the m/z-range 100–500 (1 scan/s). Inserts indicate the proposed structures of the derivative, i.e., the PFB ester, with a molecular mass of 309.2 and the most intense anion with m/z 128 for the pyroglatamate anion. Under the assumption of complete derivatization and extraction, the mass spectrum was generated from injection of 10 pmol of the derivative.

We tested the utility of the versatile derivatization reagent PFB-Br [13] for the preparation of the PFB ester of pGlu (5-oxo-proline). We used a derivatization procedure suitable for the derivatization of inorganic anions, such as nitrate and nitrite, in their aqueous acetonic solutions [22]. GC-MS analysis of the ethyl acetate extract of the derivatization reaction of synthetic pGlu with PFB-Br revealed several GC-MS peaks. The mass spectrum of the GC-MS peak with a retention time of 7.18 min (PT#1) is shown in Figure 11. The most intense mass fragment was *m*/*z* 128 ([M-PFB]^−^), which most likely corresponds to the carboxylate anion of pGlu (molecular mass of pyroglutamic acid, C_5_H_7_NO_3_, 129.1). The very weak mass fragment at *m*/*z* 196 is likely to correspond to the pentafluorobenzaldehyde. We have no indication for the reaction of the secondary amine group of pGlu with PFB-Br. Under the same derivatization conditions, glutamate, glutamine, proline and hydroxy-proline were found not to generate PFB esters extractable into ethyl acetate (data not shown).

## 4. Discussion

In GC-based methods, derivatization primarily aims to increase thermal stability and to render analytes sufficiently volatile for GC separation at high temperatures that prevail in GC-MS apparatus. Derivatization of analytes also aims to maximize the sensitivity of their detection, for instance by introducing strongly electron-capturing F atoms for ECNICI. Individual amino acids, and several dipeptides and tripeptides, were analyzed after derivatization by GC-MS in the ECNICI mode [23,24], or by GC-FID [25]. The tripeptides GSH and homoglutathione were analyzed by GC-MS using different derivatization reagents [10,11]. For the GC-MS analysis of GSH, we proposed the derivatization of GSH to its dimethyl ester pentafluoropropionyl derivative, using in situ prepared [*dimethylo*-^2^H_6_] GSH as the internal standard [12]. To the best of our knowledge, no GC-MS methods were proposed for glutathione disulfide (GSSG; MW, 612), thus far. In the present work, we demonstrate that esterification in methanolic HCl of GSH, GSSG, γ-Glu-Cys and OPH results in temporary formation of pGlu, which can be quantitated by GC-MS after subsequent derivatization with PFPA in ethyl acetate. This method is useful for the specific and quantitative determination of γ-Glu-containing oligopeptides, including GSH, GSSG, γ-Glu-Cys and OPH. The method could also be useful for the assessment of *S*-glutathionylation, i.e., proteins oxidized on their Cys moieties in the presence of GSH. Yet, this remains to be demonstrated.

Free pGlu is an endogenous substance derived from the metabolism of proline [26]. pGlu occurs in rabbit red blood cells at about 55 µM [27], in human urine at 27 µmol/mmol creatinine [28] and in human cerebrospinal fluid in the concentration range 10–35 µM [29]. Our observations suggest that free pGlu would not interfere with the analysis of pGlu in situ formed from γ-glutamyl peptides, when derivatization is performed as described in the present work. The quantitative analysis of physiological and synthetic γ-glutamyl peptides by LC-MS was recently reported [30]. It is worth mentioning that pGlu can artefactually be formed from Gln and Glu when analyzed by LC-MS/MS [21]. Massive cyclization (by up to 75%) of Gln to pGlu was observed in human serum using proton nuclear magnetic resonance (^1^H NMR) spectroscopy [31]. In our methods, Glu, proline and hydroxyproline do not interfere with the analysis of pGlu in situ generated from γ-glutamyl peptides.

**Scheme 1:**
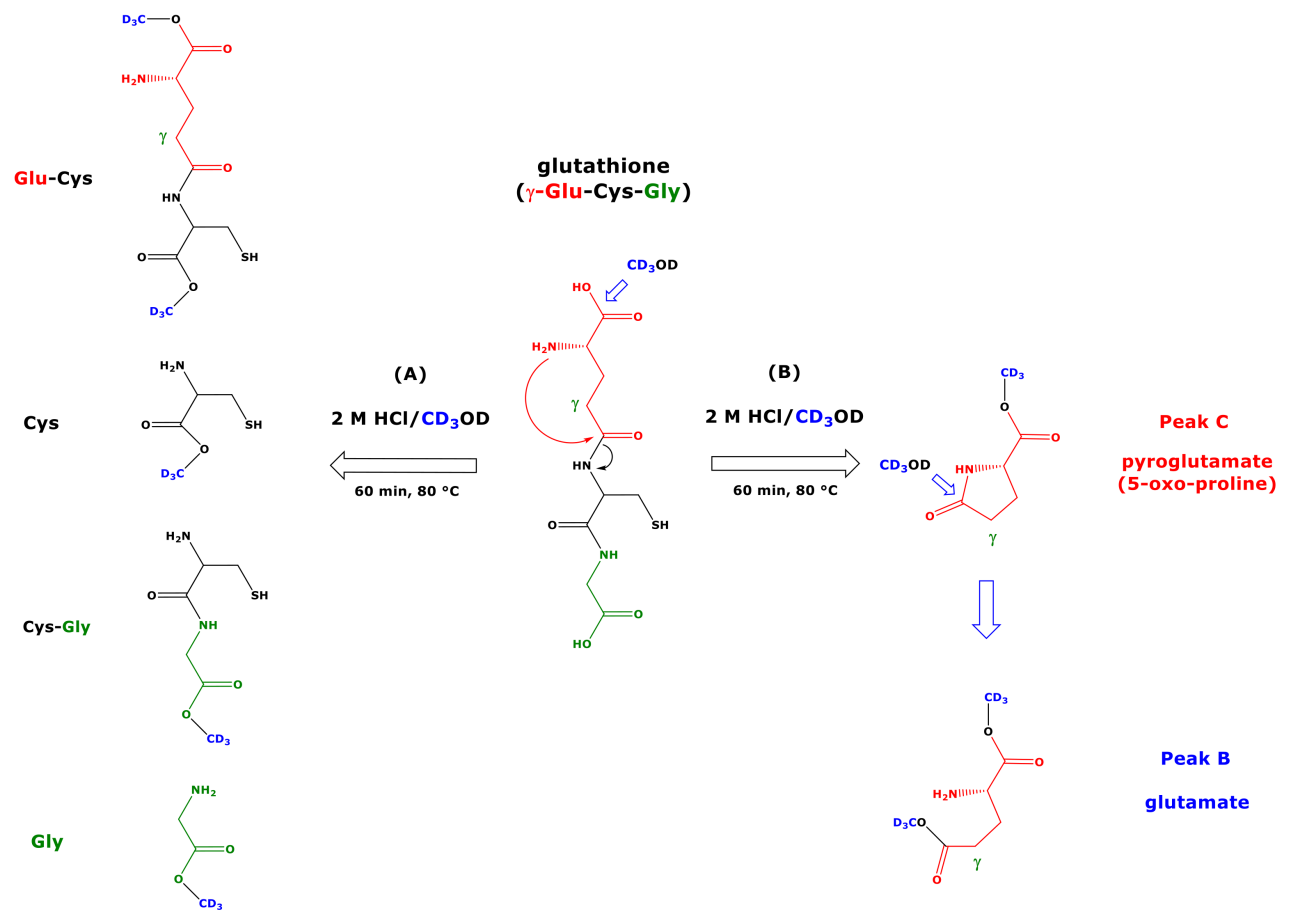
Proposed reaction products and mechanisms leading to the formation of the indicated (**A**) amino acids and dipeptides, and for (**B**) pyroglutamate upon the reaction of glutathione with 2 M HCl in deuterated methanol for 60 min at 80 °C. Peak B and peak C refer to Figure 1.

Derivatization of GSH, first with 2 M HCl in CH_3_OH, and then with PFPA in ethyl acetate, generates several derivatives due to the hydrolysis of GSH to Cys, γ-Glu-Cys, Cys-Gly and to pGlu (Scheme 1). Derivatization of GSSG with 2 M HCl in CH_3_OH also leads to pGlu formation. The derivatization method is useful for the analysis of OPH as pGlu. As OPH lacks Cys moiety, the formation of pGlu from derivatized GSH and OPH does not require a Cys moiety next to the γ-Glu moiety. Quantitative determination of γ-glutamyl species, including GSH, GSSG and asymmetric glutathione disulfides with proteins, is possible by this method because parallel derivatization of γ-glutamyl species with 2 M HCl in CD_3_OD takes place under the same conditions and yields deuterium-labeled pGlu for use as an internal standard.

The two-step derivatization procedure reported in this work is generally applicable to selectively convert N-terminal γ-glutamyl peptides to pGlu, and to quantitate them by stable-isotope dilution GC-MS. The two-step derivatization procedure can also be used to discriminate GSH from GSSG and, presumably, from high-molecular-mass γ-glutamyl proteins. To the best of our knowledge, the derivatization procedure that uses PFB-Br is specific to free pGlu and was not reported so far. The PFB-Br-based procedure requires the use of commercially available stable-isotope labeled pGlu analogs as internal standards for quantitate analyses by GC-MS. The derivatization procedures reported in this work are considered complementary and should be useful in the analysis of γ-glutamyl peptides and free pGlu by GC-MS, and possibly by LC-MS/MS.

A possible mechanism for the formation of the methyl ester of pGlu from GSH, during its derivatization with 2 M HCl in deuterated methanol (CD_3_OD), is outlined in Scheme 1. The acid-catalyzed esterification of the carboxylic group of the γ-glutamyl moiety of GSH is accompanied by the attack of the primary γ-amine group on the amide group between the γ-glutamyl and the cysteinyl moieties to form a lactam, i.e., the methyl ester of pyroglutamate (d_3_Me-pGlu). The lactam moiety of d_3_Me-pGlu reacts with another CD_3_OD molecule, which opens the ring, thereby forming d_3_Me-Glu-d_3_Me or (d_3_Me)_2_-Glu, i.e., the dimethyl ester of Glu. Our study suggests that (d_3_Me)_2_-Glu is not converted into d_3_Me-pGlu, indicative of a higher reactivity of the amide group between the γ-glutamyl/cysteinyl group of GSH compared with that of the methyl ester of pGlu. Such a mechanism is supported by observations that, under very similar reaction conditions, glutamine, asparagine and citrulline are converted to the methyl esters of glutamate, aspartate and ornithine, respectively [14].

We applied the present GC-MS method to analyze GSH in gastric mucosa of untreated rats (group 1) and rats treated with iodoacetamide (IAA) (group 2, group 3), a sulfhydryl (SH)-group-specific reagent and reported the results earlier [32]. We did not find statistically significant differences (one-way ANOVA) between the three rat groups with respect to pGlu. The following PAR values of unlabeled-to-labeled analytes were observed: 5.2 ± 0.9, 5.0 ± 1.6 and 5.9 ± 1.2 (*p* = 0.49) for pGlu; 21 ± 5, 23 ± 5, 26.7; (*p* = 0.31) for Glu; and 4 ± 0.4, 4.8 ± 1.7, 4.5 ± 0.5 (*p* = 0.45) for the Glu-to-pGlu ratio, respectively.

## 5. Conclusions

Under the esterification conditions (2 M HCl, methanol; 80 °C), GSH, GSSG, Glu-Cys and OPH form the methyl ester of pyroglutamate (Me-pGlu). Subsequent derivatization with PFPA (65 °C) forms the Me-pGlu-PFP derivative. This two-step derivatization method is useful for the specific measurement of γ-glutamyl peptides from animals (GSH, GSSG, OPH) and, presumably, from plants (phytochelatins) in various areas of research, including oxidative stress. The one-step derivatization with PFB-Br is specific to the GC-MS analysis of free pGlu as a PFB ester. These derivatization procedures should be useful for qualitative and quantitative analyses of GSH and OPH by GC-MS in biological samples, using optimized conditions and thorough method validation, including intra- and inter-assay precision and accuracy in the respective biological samples.

## Data Availability

The study did not report any data.

## Sample Availability

Not available.
